# Novel concept suppressing plasma heat pulses in a tokamak by fast divertor sweeping

**DOI:** 10.1038/s41598-022-18748-x

**Published:** 2022-10-11

**Authors:** J. Horacek, S. Lukes, J. Adamek, J. Havlicek, S. Entler, J. Seidl, J. Cavalier, J. Cikhardt, V. Sedmidubsky

**Affiliations:** 1 Institute of Plasma Physics of the CAS, Za Slovankou 3, 182 00 Prague 8, Czech Republic; 2 FNSPE, Czech Technical University, Břehová 7, 115 19 Prague, Czech Republic; 3 FEL, Czech Technical University, Technická 2, 166 27 Prague, Czech Republic; 4 ETH, Zurich, Switzerland

**Keywords:** Nuclear fusion and fission, Magnetically confined plasmas

## Abstract

One of the remaining challenges in magnetic thermonuclear fusion is survival of the heat shield protecting the tokamak reactor vessel against excessive plasma heat fluxes. Unmitigated high confinement edge localized mode (ELM) is a regular heat pulse damaging the heat shield. We suggest a novel concept of magnetic sweeping of the plasma contact strike point fast and far enough in order to spread this heat pulse. We demonstrate feasibility of a dedicated copper coil in a resonant circuit, including the induced currents and power electronics. We predict the DEMO ELM properties, simulate heat conduction, 3D particles motion and magnetic fields of the plasma and coil in COMSOL Multiphysics and Matlab. The dominant system parameter is voltage, feasible 18 kV yields 1 kHz sweeping frequency, suppressing the ELM-induced surface temperature rise by a factor of 3. Multiplied by other known mitigation concepts, ELMs might be mitigated enough to ensure safe operation of DEMO.

## Thermonuclear fusion reduction due to ELM-induced tungsten droplets

**Figure 1:**
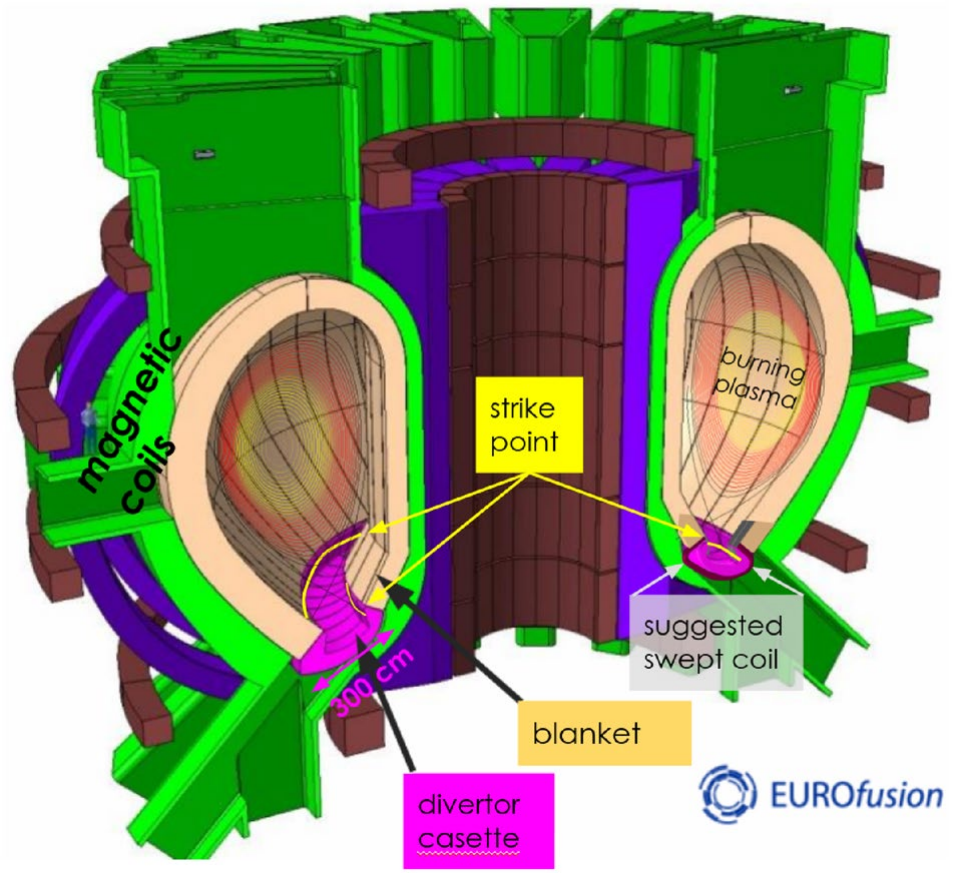
Sketch of DEMO tokamak^5^ depicting the most heat loaded divertor plasma contact *strike point*. In poloidal cut it’s a very localized point, whilst in 3D it’s a toroidal circle. Here we suggest to sweep it by a coil up/down by $\lambda _{\mathrm {swp}}$=7 cm whilst the divertor cassette is 300 cm large.

Thermonuclear fusion reactors represent a promising clean, safe, sustainable 24/7/365 and economical^1^ energy source, however, still eight major challenges^2^ need research attention. One of them is survival of the internal heat shield against steady and pulsed heat plasma loads. This is feasible on most of the in-vessel components^3,4^ loaded by plasma with typical temperatures around 10$^{4-6}$ Kelvin and density 10$^{6-7}$ lower than the ambient air, yielding thus 1–100 MW/m$^2$ level heat flux. Especially at the divertor strike point area (Fig. 1), where plasma unfortunately strongly concentrates by the toroidal magnetic field, its survival is a challenge.

The ITER tokamak is currently the most expensive science experiment on the planet^6^. Its goal^7^ is to demonstrate 500 MW steady-state repetitive thermal fusion power production starting in 2035, reliably operating with non-burning plasma since 2025. Since for ITER it’s already too late to plan installation of coils required for this novel concept, we focus to demonstrate its usefulness for its successor (see Table 1): EU DEMOnstration power plant with construction start before 2040^2^.

Already in ITER low energy confinement L-mode without any fusion power, plasma heat flux component perpendicular to the heat shield surface $q_\perp =$10 MW/m$^2$ is predicted^8^ in attached divertor plasma. This is comparable to a rocket combuster nozzle^9^, p.320]. In a plasma with a fusion gain Q=10 in the high-confinement (H)-mode, much stronger heat flux is expected. Artificial increase by *impurity seeding*^10^ of natural edge plasma radiation cooling (called detachment) is necessary, however, without undesired cooling of the plasma core or dilution of the plasma itself. Radiation increase from 30% upto 93% would be necessary to get below the expected engineering limit of $q_\perp =$16 MW/m$^2$ of the best water cooled tungsten heat shield (figure 18 in^11^). It is limited by the surface water boiling which was, by the way, just recently overcome^12^. Testing survival under those extreme conditions (mostly using liquid metal heat shield^13^) will be one of two major goals of tokamak COMPASS-Upgrade^14,15^, capable of reaching $q_\perp \approx$100 MW/m$^2$^16^. This is, for better imagination, comparable to the Sun surface 63 MW/m$^2$ radiation. Alternative divertor geometries are also intensively studied^17,18^ as an exhaust solution.

On top of this, H-mode plasma unfortunately generates short intensive *heat pulses*: accidental sudden loss of divertor *detachment*^10^ and regular edge localized modes (ELMs)^19^. ELM energy is mitigated by vertical kicking^20^, resonant magnetic perturbation (RMP^21^) or pellet injection^22^.

**Table 1:** Overview of mentioned tokamaks.

Tokamak	Country	Start year	Plasma energy [MJ]	Fusion power [MW]
AUG	Germany	1991	1	0
COMPASS-U	Czechia	2025	1	0
JET	EU	1974	8	11
SPARC	USA	2028	24	140
ITER	Int’l.	2035	500	500
DEMO	EU	2060	1300	4200

At top: copper coils limit discharges to less than 10 s. At bottom are superconductive tokamaks with much longer operation. Energy released within regular ELMs is a few % of the plasma energy at frequency $10^{0{-}2.5}$ Hz.

ELM delivers huge energy (projected to the surface normal $\epsilon _\perp =$1.2 MJ/m$^2$ predicted for ITER^23,24^) strongly localized around the divertor strike point within a millisecond-long pulse. This implicates the tungsten surface to heat well above recrystallization (which strongly limits the cracking lifetime^25^) and even flash-melting^26,27^. For the SPARC tokamak, ELMs are predicted to pulse $\epsilon _{\perp }\approx 2$ MJ/m$^2$^28^. $\epsilon _\perp =10$ MJ/m$^2$ is similarly predicted for the EU DEMO^29^.

So far, only a few successful ELM mitigation experiments were worldwide achieved by argon seeding (e.g. by 60% in^30^). Without ELM mitigation, the simulation^31^ predicts certainty of melting and ablation at the tile *edges* already above 0.15 MJ/m$^2$ due to finite ion orbit drifts. ELM triggering^22^ by pellet injection has questionable reliability, yet far below 100%. Just a few unmitigated ELMs yields a run-away damage of its edges^32^ due to rise of the plasma-surface incident angle at places where the molten tungsten re-solidifies^26,27^ after having slipped along its surface, pushed by thermoelectric current^33^.

Relatively newly adopted, enhanced $D_\alpha$ EDA or quiescent QH or radiative-improved I modes are therefore intensively studied. However, they still expel plasma in some kind of regular bursts (e.g. the pedestal relaxation events), just a few times weaker than Type-I ELMs^34, Fig. 6^. The here-proposed fast strike point sweeping can likely be successfully applied on all those various bursts.

Survival of heat shield made from liquid metals wetted into a capillary porous structure, loaded by such ELM-like pulses, has been demonstrated on non-tokamak devices (see Table 1 in^35^) and for the first time directly in a tokamak ELMy H-mode divertor^36^. However, the plasma contamination by huge SnLi vaporization and sputtering will pose another challenge to overcome.

Regardless, the baseline scenario for ITER is still the H-mode with Type-I ELMs on solid tungsten divertor. Even though for DEMO ELM-free high-confinement scenarios are being intensively developed^37^, ELMs are extremely useful for two reasons:

- ELMs keep the plasma under the density limit. Density accumulation was the main reason why in 1997 in JET ELM-free scenario the record fusion power 16 MW was limited to only 0.5 s^38^. Therefore, additional resonant magnetic perturbation (RMP) coils are intensively studied as they seem to stabilize the plasma core and offer sustainable ELM-free regime
- ELMs expel accumulated impurities (tungsten, O$_2$ and helium ash from the DT burn) from the plasma edge^39^. Tungsten core accumulation was exactly the main reason why (expectedly) the historic achievement of 59 MJ fusion energy record^40^ (repetitively produced in 2021 in JET) was not significantly higher than 11 MW.

It thus seems that at least ELMs with high frequency and low energy are therefore unavoidable, however, the subsequent heat impulse on the heat shield has to be mitigated at least below tungsten melting 3422$^o$C (releasing droplets) or even better recrystallization (around 1600$^o$C) releasing dust by surface cracking and strongly shortening its lifetime.

Core plasma accumulated tungsten is a problem because even tiny concentration $3\times 10^{-5}$ increases by 20% the fusion $nT\tau _\mathrm {E}$ limit (due to strong plasma cooling by line and bremsstrahlung radiation), only for which a thermonuclear burn is possible^41^. This corresponds to a single millimetre-size tungsten droplet penetrating into the core of DEMO-size tokamak. Ejections of a few droplets per second (each with fortunately 10$^3\times$ smaller volume) were indeed observed in JET from a single deliberately-exposed tungsten divertor lamella edge melted by ELMs^26,27^. On JET, the impact on the plasma was fortunately minor thanks to just tiny melted area. In ITER and DEMO, however, regular melting of not only the edges but of the entire divertor area ($10^3\times$ larger than the single JET lamella) is expected if ELMs will not be sufficiently mitigated.

Therefore, a novel physics concept is here proposed, capable to mitigate the ELM-induced surface temperature damage^42^ of solid divertor targets. It sustains of spreading the heat flux by harmonic sweeping of the divertor strike point, using a dedicated divertor coil in a simple resonant electrical circuit with a powerful capacitor bank at high voltage. Sweeping fast and far enough during this regular millisecond event, the heat spreads over larger surface.

Since ELM-mitigating effects of the impurity seeding^10^, the ELM frequency control^17,39^, the alternative divertor geometries^18^ and this here-proposed fast strike point sweeping all somehow multiply together, safe operation of high confinement plasma in future fusion reactors may be possible, however, clearly requiring intensive further research of their mutual interaction.

## Fast strike point sweeping

A dedicated in-vessel divertor magnetic coil could reliably provide such fast sweeping. The more localized magnetic field, the better since it would require the weakest power supply, cooling and would yield the lowest undesired influence on the confined plasma. We suggest a simple harmonic oscillation circuit where the frequency $f^{-1}_{\mathrm {swp}}=2\pi \sqrt{LC}$ is determined by the divertor coil inductance *L* and a capacitor *C*. Interestingly, the bigger the tokamak, the more cost-effective and important this technique might be because

- the ELM heat distributes on an area independent from the tokamak size^23^, thus relatively more localized
- there’s more available space for the coils,
- the divertor magnetic field lines bend by this swept coil over longer distance between the X and strike points.

In contrast, higher poloidal magnetic field $B_\theta \propto$ $I_p/\pi a^2$ in future thermonuclear tokamaks requires proportionally larger $I_\text {coil}$.

Note that this proposed fast sweeping must be combined with a slow sweeping: in Fig. 1 in^43^ a slow sweep with 4 Hz and 200 mm is already assumed. Slow sweep is a relatively standard technique used also sometimes on the tokamak JET. This is necessary for three reasons:

- enlarge the deposited area of the steady inter-ELM plasma heat flux
- shift each subsequent ELM deposition area to another region
- avoid overheating of the (subsurface pipe) cooling water, as simulated for DEMO^44^

Slow sweep (Hz) is, however, much easier than the fast (kHz) sweep. Below the vessel self-frequency (for DEMO it’s 9 Hz^43^), such slow sweep would be provided by the external (outside the vacuum vessel) superconductive coils. Its slowly varying magnetic field penetrates inside the vessel. However, kHz sweep would be fully suppressed by the induced vessel eddy currents. Therefore only in-vessel divertor coils are considered for the fast sweeping, made of copper to avoid neutron-irradiation problems of superconductors.

This paper simulates feasibility of this yet-untested concept, merging together many issues (detailed in^45^). First, we predict the ELM plasma properties for the far-future DEMO reactor. Then, heat conduction Matlab simulation yields the required space and time sweeping amplitude, performed by alternating strong magnetic field of a dedicated coil interacting with the plasma, including the induced eddy currents in surroundings. This required the alternating magnetic conductor, accounting for the plasma particle dynamics, all simulated in COMSOL Multiphysics and Matlab. Finally, theoretical feasibility of the required high power electronics is quantified.

## DEMO ELM space-time scale & heat conduction for prediction of $F_{\mathrm {STS}}$

**Figure 2:**
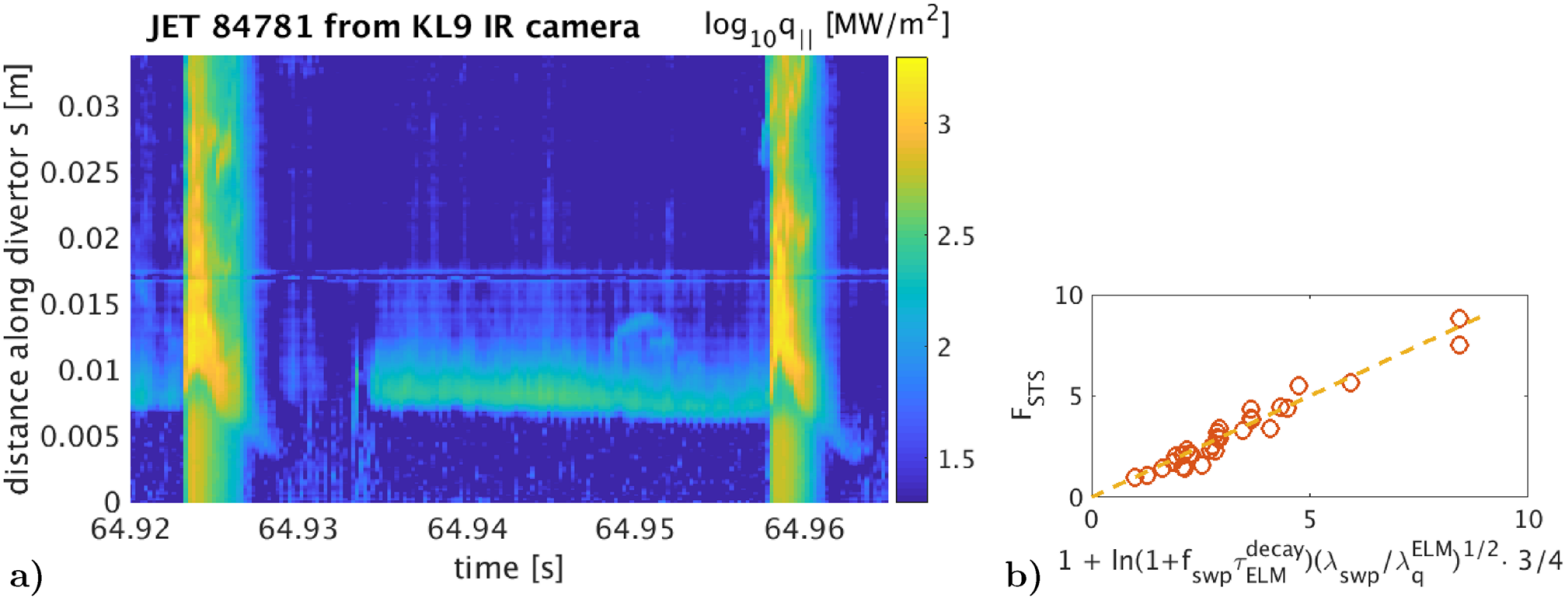
(**a)** The plasma divertor heat flux space-time distribution from JET, used in^43^, already after being rescaled (i.e. multiplied in time by 1.2 and in space by 0.28). Broadening of the heat flux spatial profile from inter-ELM to ELM is clearly visible. Exponential decay fit of the ELM peak in both space and time yields $\lambda _q^{ELM}\approx 2$ cm and $\tau _{\mathrm {ELM}}^{\mathrm {decay}}$=3 ms. (**b)** The additional proposed sweeping of the divertor *s* strike point yields to this empirical scaling of $F_{\mathrm {STS}}$  found from the heat conduction simulation^43^ inside a solid target with varying $\lambda _{\mathrm {swp}}<$20 cm harmonic amplitude and $f_{\mathrm {swp}}<10$ kHz frequency.

Let’s quantify the basic system parameters: what must be the sweeping frequency and amplitude in order to suppress the ELM heat pulse significantly? From the plasma physics point of view, this we already quantified in^43, Fig.1^ by a 2D dynamic heat conduction simulation using real infra-red data of large Type-I ELM heat fluxes on the tokamak JET divertor target, simply rescaled towards the EU-DEMO1 reactor ($B_0 = 6~ \mathrm {T},~I_p = 21~ \mathrm {MA}, R_0 = 9~$m). The input data varied in space and time during both intra-ELM and inter-ELM shown in Fig. 2a. There are only two scaling factors: the ELM-induced heat flux on DEMO was assumed as identical to JET, except simply multiplied in space by 0.28 (squeezed) and in time by 1.2 (slightly prolongated) , argued in Section 2 in^43^. Note that even though absolute magnitude (rescaled for DEMO) of the heat pulse, as well as the choice of the solid target material, determines the maximum surface temperature, it plays no role for the relative suppression $F_{\mathrm {STS}}$, i.e. how many times the temperature rise gets suppressed.

This heat conduction simulation of various Type-I ELMs yields a scaling in Fig. 2b (slightly improved from^43^) of the *Surface Temperature Suppression Factor* $F_{\mathrm {STS}}$of the ELM-induced surface temperature rise (considering all peaks over the entire area).

Since the most critical engineering parameter is the maximum available voltage $U_0$ from the capacitor bank, we further fix $U_0=18~$kV as feasible from the electro-engineering point of view noting only weak dependency $F_{\mathrm {STS}}\propto \root 3 \of {U_0}$. Note that this simulation takes into account all the ELM filaments details and statistical spreading of several consecutive ELMs. The surface temperature drops back to the pre-ELM value long before another ELM arrives. Even though the sweeping introduces additional surface temperature oscillation with $f_{\mathrm {swp}}\sim$kHz, its amplitude is only a few dozens of Kelvin. This we consider negligible for its lifetime, especially if it stays below the recrystallization level. A thermal fatigue analysis should be done in future.

Due to harmonic sweep $position\propto I_\mathrm {coil}=I_0\sin (2\pi f_\mathrm {sweep}t)$, the strike point stays at the sweep *edges* longer than in the middle position ($I_\mathrm {coil}=0$) which yields undesirably low $F_{\mathrm {STS}}$  (which we do account for). During one ELM, however, the heat dissipation progressively decreases $I_0$ down to $\sqrt{1-\tau _{\mathrm {ELM}}^{\mathrm {decay}}R_\mathrm {eff}/L}$ (quantified further in Table 2) which may improve the system effectiveness because the sweep edges slowly shift. The heat conduction simulation (Fig. 1 in^43^) concludes (for sweep amplitudes smaller than 10 cm and dissipation less than half, always desired), however, that this effect is unfortunately negligible.

**Figure 3:**
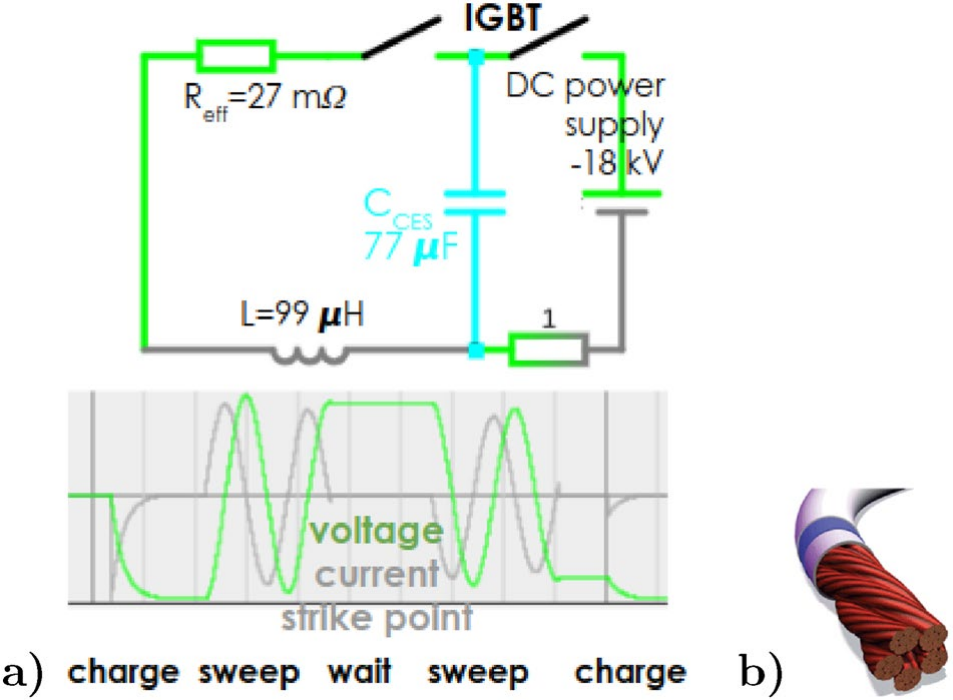
(**a**) Oscillation circuit of the swept coil. The current $I_\mathrm {coil}$ (directly proportional to the strike point position) oscillates during 2 subsequent ELMs. Charging after each even ELM suppresses the coil mechanical vibrations down to 0.1 mm due to heavy coil inertia (explained in Section 5 in^43^). (**b**) Sketch of 45 mm thick Litz conductor sustaining of twisted 0.25 mm thin wires without isolation. Such a cable suppresses the skin-effect, passing thus 1 kHz AC currents with resistivity near its DC value. Let a cooling pipe be located in the central empty space.

The strike point must not, however, oscillate all the time. After $\tau _\mathrm {ELM}=3~$ms the ELM heat pulse drops below the pre-ELM value, thus it’s not necessary anymore. This saves the capacitor energy, the need for coil cooling and permits the capacitor bank to be charged back up during the inter-ELM periods as depicted in Fig. 3a. Therefore, the capacitor must be triggered by an analogue divertor ELM-detection signal, provided probably easily by rise of a divertor grounded tile current or a (much smaller) grounded divertor probe $I_0$; possibly backed-up by D$_\alpha$, impurity line radiation or IR camera. It thus sweeps only 3 ms per each ELM with (vaguely known) waiting time $\sim$100 ms=$f_\mathrm {ELM}^{-1}$.

## 3D magnetics simulations

**Figure 4:**
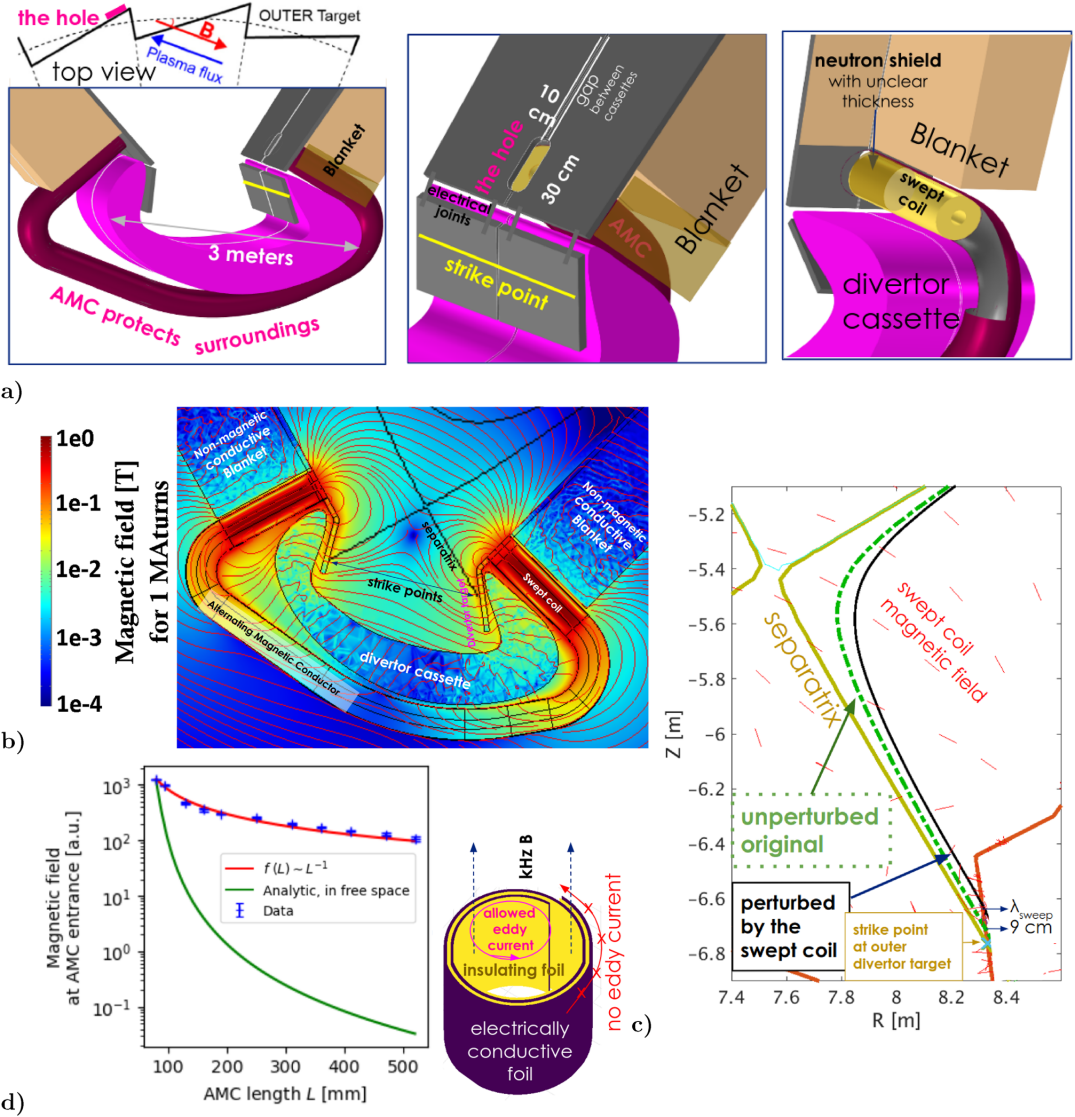
(**a**) Halves of two neighbouring heat shield DEMO divertor cassettes from Fig. 1 bottom with the hole in between, magnetically shielded by fish-scale toroidal shaping (sketched on top left, according to^47^). AMC is connected to the divertor cassette, nested into the blanket module. The vertical *electrical joints * stop the AC magnetic field from returning back to the coil. The bottom part of AMC is unnecessary (its removal does not significantly change the desired influence on the plasma), serves only to protect the surrounding diagnostics from the kHz B-field. The simulation shows that 2 cm toroidal gaps between the heavy cassettes (as on ITER^48^ due to engineering reasons) and 2 mm cuts in AMC (feasible as it’s a light tube) yields the strongest magnetic field penetration into the plasma. (**b**) COMSOL Multiphysics calculated magnetic field lines (for 1.6 kHz) penetrating through the divertor cassette hole. The induced eddy currents in the divertor targets and AMC stop its magnetic field returning straight back to the solenoid further end, thus penetrating deeper into the plasma. (**c**) Poloidal cross-section of a magnetic field line passing through the divertor region. Plasma progressively deviates outwards while flying through each swept coil (each with the maximum $N_\mathrm {turns}I_\mathrm {coil}=1$ MegaAmperTurns), such that the strike point integrated shift is $\lambda _{\mathrm {swp}}=9~$cm above the unperturbed ($I_\mathrm {coil}=0$) (thin) line. For $-I_\mathrm {coil}$, the shift is the same in opposite direction. Field lines outside the strike point shift by a similar distance. (**d**) Experimental profile of magnetic field *B* along coil axis inside the Alternating Magnetic Conductor. Sketched on right, the AMC shows $L^{-1}$ decay with distance, whilst in free space far away from the coil it decays as $L^{-3}$^49^.

In the published design^43^, even though the induced eddy currents inside the conductive vacuum vessel were accounted for, the metallic structure of the divertor cassettes^46^ was ignored. Since it probably screens the coil magnetic field, here we consider a better coil design: 2 solenoids in each divertor cassette (Fig. 4a). The swept coil magnetic field in Fig. 4b is poloidal, penetrating through a $10\times 30$ cm$^2$ hole between two divertor cassettes in Fig. 4a. The cassette generates eddy currents which suppress the magnetic field direct return to the swept coil. Since eddy currents cannot circulate around the hole between the (electrically disconnected) two cassettes, the magnetic field is not suppressed by the hole. This we verified both experimentally and within the *COMSOL multiphysics* dynamic simulation. We believe that such a hole in divertor is feasible for three reasons:

- The hole shall be between two neighbouring divertor cassettes where a magnetic shadow is anyway planned^47^ in order to protect the cassette leading edges ( see the top left Fig. 4a).
- Since the magnetic field intensity at the swept coil entrance is comparable to the main tokamak toroidal field (6 T), it’s somehow “sucking-in” plasma, however, from region 30 cm$~\gg \lambda _q$ above the strike point which is safe.
- The hole should therefore be filled with an electrically insulating plasma-facing material (such as boron-nitride) to protect the coil against neutrons and accidents.

The swept coil geometry complicates the physics simulation: the 3D magnetic perturbation looses toroidal symmetry with respect to^43^. Therefore, first we calculate the 3D magnetic field Fig. 4b within the large volume around the swept coil in Comsol Multiphysics.

The concept of fast sweeping suffer from the presence of induced eddy currents, which cause a significant interaction of the coils with the tokamak structure. This greatly impairs penetration of the magnetic field through the heat shield hole. Therefore, we invented, experimentally tested and used in the simulation an *Alternating Magnetic Conductor (AMC)*. It is a conductive tube sketched in Fig. 4d, suppressing the undesired eddy currents perpendicular to its axis, whilst allowing all other eddy currents. The swept coil is located inside the tube and generates magnetic field that shall not pass through a material of conductivity $\sigma$ and magnetic permeability $\mu$ due to the allowed eddy currents. For the AMC to function properly, its thickness $\delta >1/\sqrt{\pi f_{\mathrm {swp}}\sigma \mu }=2$ mm skin depth for copper and 1 kHz. In contrary to a waveguide, AMC guides magnetic field of much lower frequencies. The eddy currents thus prevent penetration of the surface-perpendicular component of the magnetic field. Static external magnetic field (e.g. the strong tokamak toroidal field) penetrates through AMC without any interaction (due to absence of a ferromagnet), especially produces no force and does not influence the AMC heat losses. Experiments have shown that the best AMC is a tube made of 2 foils: conductive and insulating, forming a spiral in cross section.

Unfortunately, the COMSOL Multiphysics simulation cannot reproduce the AMC quality. *B* drops with the distance much stronger than in the experiment, probably due to limited space resolution. This implicates that $\lambda _{\mathrm {swp}}$ for 1 MAt will in reality be somehow larger than further considered, therefore also $F_{\mathrm {STS}}$  better.

Simulating the system fully without AMC yields to $F_{\mathrm {STS}}$=2.1  and $\lambda _{\mathrm {swp}}=3.6\times$ smaller with otherwise similar system parameters (especially ohmic losses), thus significantly worse than with AMC.

We then simply sum the swept coil 3D vector field (repeated 54$\times$ toroidally) with CREATE plasma equilibrium magnetic field of the 2017 DEMO baseline^50^. In Matlab we track the magnetic field lines in 3D, its poloidal cross-section shown in Fig. 4c. This simulation yields that each field line deviates by $\lambda _{\mathrm {swp}}$ = 9 cm (quite independent from the separatrix distance, dropping with $f_{\mathrm {swp}}$) due to passing through the 54 swept coils (each with 1 MAt), as desired.

**Figure 5:**
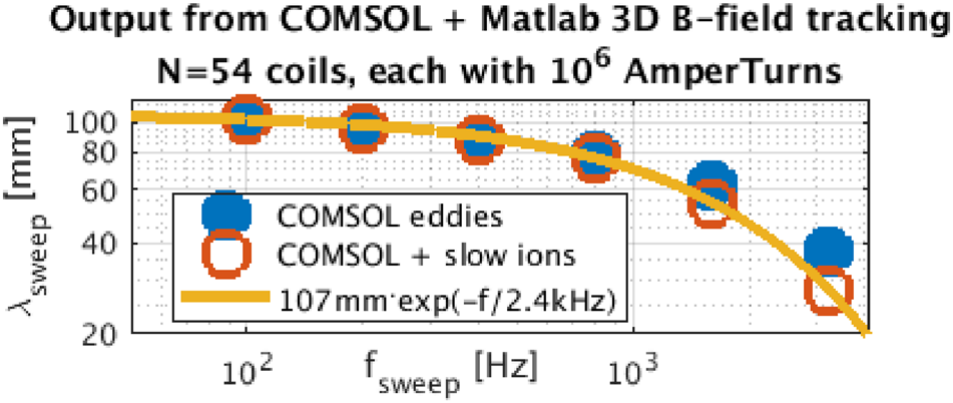
The overall desired strike point shift $\lambda _{\mathrm {swp}}$ for 10 kA$\times ~100$ turns $\times$ 54 coils (directly proportional), simulated for the most heat loaded (outer) target. Eddy currents in the divertor structure and AMC generate (undesirably) a frequency low-pass filter. Similarly, the effect of slowly flying ions further weakly decreases. The yellow fit is further used in Fig. 6 and Table 2.

Since the mutual phase shift between the coil current and the eddies increase with frequency, the resulting swept coil magnetic field shape and amplitude drops. The scan in Fig. 5 over frequencies shows that those eddy currents effectively decrease the strike point shift.

There is additionally another limit for the frequency, determined by the fly-time of ions through the swept coil regions. According^51^, $T_\mathrm {e, ELM}^\mathrm {divertor}\approx 0.8\cdot T_\mathrm {e}^\mathrm {pedestal}$. Assuming that it’s 5 keV, the fly-time of Deuterons with the corresponding $c_{s,i}=\sqrt{2T_ie/m_D}=700~\mathrm {km/s}$ through the swept coil magnetic field region is $2\pi R/c_\mathrm {s,i}\approx 100~\upmu$s because each ion passes $\sim 2\times$ toroidally around and the desired $\lambda _{\mathrm {swp}}$ integrates along this path. During 100 $\upmu s$, however, the coil current vary significantly. Taking both effects into account, for the most populated ions with $v\sim c_{s,i}$, the strike-point sweeping amplitude drops with frequency as in Fig. 5.

**Figure 6:**
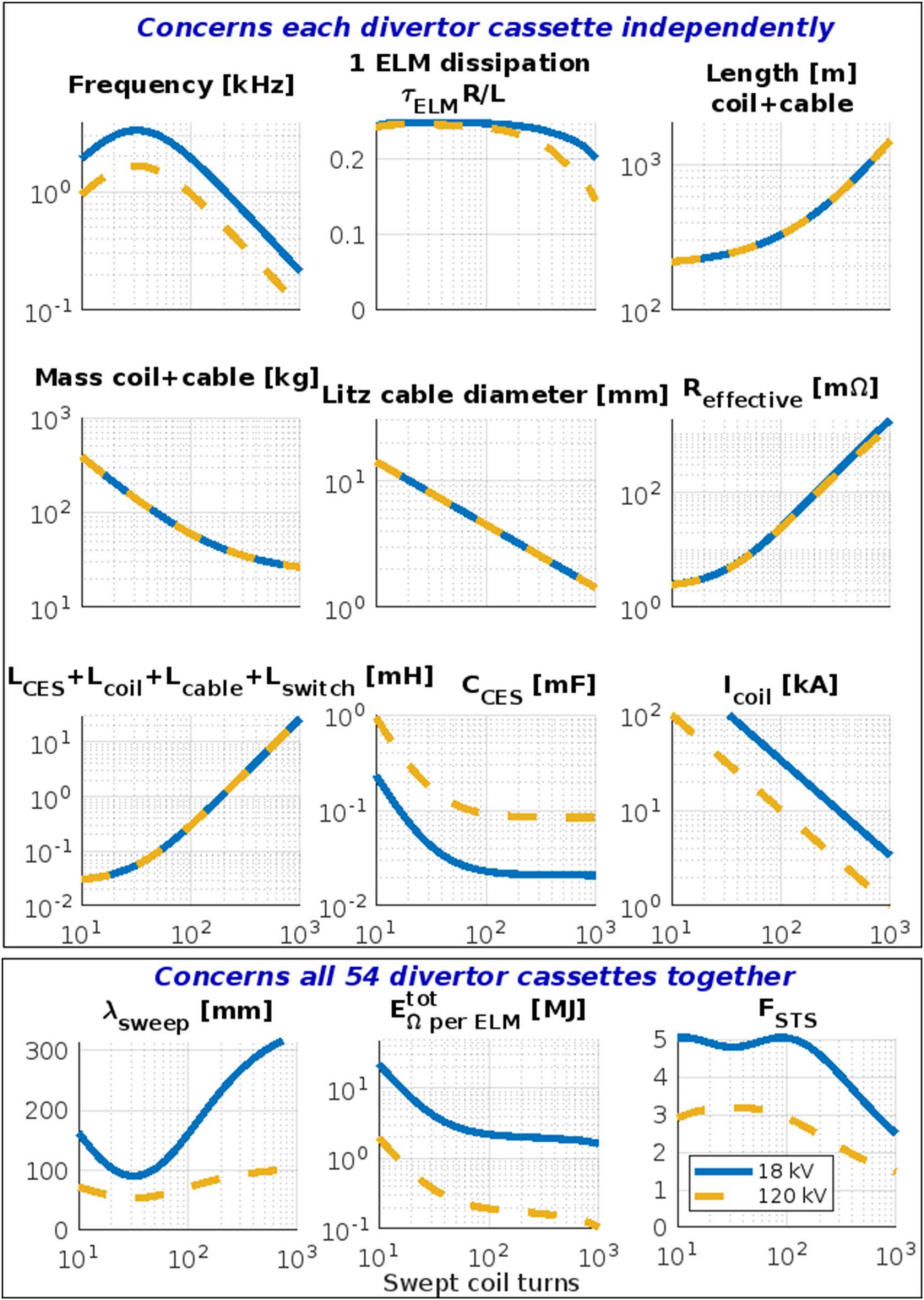
Scan over number of the swept coil turns. Assuming fixed geometry of the swept coil in DEMO divertor, 200 m distance to the capacitors, N = 54 coils, $\lambda _{\mathrm {swp}}$ from Fig. 5, $U_0$ = 18 (blue) $\Vert$ 120 (yellow) kV, $C_{cable}$ = 1.6 nF. $L_{cable}$ = 28 $\upmu$H. $\tau _{\mathrm {ELM}}^{\mathrm {decay}}$ = 3 ms, $\lambda _q^\mathrm {ELM}$ = 2 cm. $L_\mathrm {CES}=25~n$H, $R_{CES}=0.1~m\Omega$.

The system quantities are interconnected through the relations at the top of Table 2, quantified in Fig. 6. While scanning over number of the turns, we keep $N_\mathrm {turns}\cdot I_{coil}$ constant and assuming feasible connection through 100 meters long Litz double cable to each swept coil. We also keep constant the coil geometry, volume and mass. For $N_\mathrm {turns}\gg 100$, the connection to the capacitor plays negligible role, however, $F_{\mathrm {STS}}$ drops due to slower resonant frequency $f_{\mathrm {swp}}\propto 1/N_\mathrm {turns}$. For $N_\mathrm {turns}\ll 100$, the ohmic heating dissipation loss and total mass (including thicker long Litz cable) rise. Ohmic losses of eddy currents in surroundings (calculated in COMSOL Multiphysics) are stronger than those in the cable and coil and they rescale as $E_\Omega ^\mathrm {eddy}\propto \sqrt{f_{\mathrm {swp}}} I_\text {coil}^2$. Therefore, energy lost during one ELM $E^{\mathrm {tot}}_{\Omega \mathrm {/ELM}}$  drops with $N_\text {turns}$. Relative (capacitor) energy dissipation during one ELM is

$$\begin{aligned} \frac{E^{\mathrm {tot}}_{\Omega \mathrm {/ELM}}}{E}= \frac{R_\text {eff}}{L}\int _0^{\tau \text {ELM}}\sin ^2(2\pi f_{\mathrm {swp}}t)e^{-2tR_\text {eff}/L} dt. \end{aligned}$$

which has a complicated analytical solution.

The highest $F_{\mathrm {STS}}=3$ with acceptably low $E^{\mathrm {tot}}_{\Omega \mathrm {/ELM}}$  and the copper mass is reached for $N_\mathrm {turns}\sim 100$, for which the crucial system parameters are summarized at the bottom of Table 2, only weakly dependent on all the above assumptions. The Surface Temperature Suppression Factor rises weakly with voltage as $F_{\mathrm {STS}}\propto \root 3 \of {U_0}$. Varying $\lambda _{\mathrm {swp}}$ yields

$$\begin{aligned}&E^{\mathrm {tot}}_{\Omega \mathrm {/ELM}}\propto \frac{1}{2}C_\mathrm {CES}U_0^2=\frac{1}{2}LI_0^2\propto \lambda _{\mathrm {swp}}^2\\&U_0\propto \mathrm {velocity^{strike}_{point}}= 2f_{\mathrm {swp}}\lambda _{\mathrm {swp}}\sim F_{\mathrm {STS}}. \end{aligned}$$

Choosing $\lambda _{\mathrm {swp}}\approx 7$ cm yields thus acceptably low $E^{\mathrm {tot}}_{\Omega \mathrm {/ELM}}$=2.6 kJ per coil with low enough $f_{\mathrm {swp}}$.

Higher $F_{\mathrm {STS}}$=5 could be reached, however, for much more demanding hardware: higher $U_0=120$ kV, $I_0=33$ kA, $\lambda _{\mathrm {swp}}=16~$cm and especially $E^{\mathrm {tot}}_{\Omega \mathrm {/ELM}}=1.9$ MJ which we consider unacceptably high for e.g. 10 Hz ELM frequency (thus 19 MWe input for the entire system).

We consider all the coils connected as *fully independent circuits* due to safety: if several (*n*) of them break down, the system still works, with just proportionally lower amplitude $\propto \frac{N-n}{N}$. Another (unsafe) option would be to connect them all parallel into a single circuit with 54$\times$ bigger CES. However, our market search concluded that such a big CES would anyway sustain from a similar amount of components, thus would not yield any advantage.

## Feasibility of high power electronics

A kHz frequency current cannot be driven through a normal thick copper cable due to the skin effect. However, *Litz wire* (Fig. 3b) with non-insulated strands, which we consider as a necessary condition in neutron radiation environment, has only 5$\times$ higher $R_{AC}/R_{DC}-1$ increase with frequency^52^ with respect to an insulated Litz wire. At frequency below 100 kHz, however, $R_{AC}/R_{DC}\sim 1$. Therefore, at our frequency 1 kHz and the strands thinner then 0.25 mm, we may still assume $R_{AC}\approx R_{DC}$. Swept coil Litz-type conductor 36 mm thick thus yields resistivity $R_\text {AC}$ low enough to dissipate only 54$\cdot$2.6 kJ per ELM. Assuming ELM frequency e.g. 10 Hz^11^, this means 1.4 MW of total ohmic losses. Assuming e.g. cooling by 1 liter/s water flow, the outlet water heats up by 6 Kelvin which we consider well acceptable.

Figure 6 demonstrates that the capacity of the *Capacitor Energy Storage (CES)* is the loosest parameter and is in the order of commonly available CES. So a standard non-polarized CES, which can work in AC mode, will suffice. Since $F_{\mathrm {STS}}\approx \root 3 \of {U_0}$, the largest possible voltage is desired. On the other hand, the dependence is so weak that we prefer to limit ourselves to a voltage of $\sim 18$ kV, under which there are still no undesirable problems with sparking in the circuit. We consider ring-film independent capacitor (8 kV, 50 $\upmu F$) in series of 5 in 10 parallel channels, which yields a voltage of 20 kV with a capacity of 0.1 mF, a parasitic resistance of $R_\mathrm {CES}=$0.1 m$\Upomega$ and a parasitic inductance of $L_\mathrm {CES}=$25 nH for a single coil.

For *switching ON/OFF* the above mentioned voltages and currents, let IGBT transistors be used. Since any available discrete semiconductor switches have insufficient breakdown voltage at maximum current, we consider a Series-Parallel (15$\times$6) combination of the IGBT transistors meeting our requirements for a 18 kV and a maximum continuous current of 22 kA, which is sufficient for one of the 54 coils. Since the voltage drop is only 2 V for one ON switch, we get an effective resistance of $15\cdot 2~{\text{V}}/10~{\text{kA}}$=1.4 m$\Upomega$ for one coil. The AC circuit requires two opposing switches where each takes current in one direction. In total, the system thus requires $54\cdot 15\cdot 6\cdot 2=9720$ IGBT transistors (weight of each is 2 kg). Their parasitic capacitance are negligible with respect to the capacitor, as well as the capacitor resistivity is negligible with respect to the switch. An example of the feasibility of such a switching approach is the industrial pulsed power device presented in^53,54^.

Concerning *lifetime*, 2 full power years operation is planned for the cassettes exchange period, determined mostly by the neutron irradiation^46^. In the region where the coil is located, outside the water cooled divertor cassette, it will be less by several orders of magnitude, around $10^{-1}$ DPA displacement/atom/year. Therefore the lifetime is probably not a problem for the coil (including a 0.1 mm amplitude vibration^43^, Section 5]), cables, capacitors and the simple ELM-detection diagnostic (the divertor tile grounded current detection). This means, however, nearly a billion ELMs, each requiring a single ON/OFF switch of ±18 kV at 0 kA and passing ±10 kA AC current for 3 ms with 100 ms cooling time. If the IGBT transistors (100 m away from the tokamak) will be well cooled and well protected against over-current or over-voltage, the assumed $10^9$ number of cycles to failure is achievable already with nowadays technology^55^.

## Disclaimers and discussion

Several feasibility issues were not treated here due to its complexity and probably relative insignificance. Especially, the integration of this system into the DEMO design would have to be assessed together with the DEMO Central Team before deciding on its possible use there.

Most probably, the plasma-generated magnetic field sum with the swept coils and eddies would not only generate the linear perturbation (used in the 3D magnetics tracking) but also *ergodic regions* (with fully chaotic 3D topology) similar to the *resonant magnetic perturbation (RMP)*^56^ technique. We think, however, that the *ergodicity in our system is much weaker* than in RMP because of

- much higher toroidal symmetry (toroidal number of coils $N=54\gg 9$ considered for ITER RMP^56^),
- all the swept coils have the same polarity whilst in RMP the even/odd coils switch polarity,
- RMP coils located at midplane integrate the chaotic perturbation over much longer distance than the swept coils in divertor.

Therefore, in this study we ignored the ergodicity issue.

We also ignore the *skin effect from induced currents in the plasma* by this swept coil oscillating magnetic field. In similar calculations for RMP^21^, these plasma-induced currents somehow *reduce*^56^ the RMP-field penetration into the plasma. We did not consider this issue due to its complexity and it should be investigated in follow up studies.

We also don’t consider core plasma MHD instabilities which might be induced by this kHz magnetic field, however, we think it’s negligible because a) the perturbation is strongly localized within the divertor region, b) it’s present *only* during the ELM crash, not otherwise.

Compared to the published^43^ design, the resulting system requirements are similar. The reason is that the necessary magnetic energy $\int B_{\mathrm{coil}}^2\mathrm{d}V$ of the required magnetic field line perturbation and its time-scale are similar, even though very different in shape, volume and amplitude. Unfortunately, here we predict the sweeping performance significantly lower due to the eddy currents.

*Liquid* metal divertor (LMD) target is a concept overwhelming the *solid* metal (tungsten) due to much better survival of the extreme heat pulses and fluxes (Table 1 in^35^). Its main drawback is, however, much stronger plasma impurity contamination which requires significant further research. Even though LMD sustained well the strongest ELMs on tokamak COMPASS^36,57^, sweeping above *liquid* target is predicted *not* to be useful^13^ because the target surface cooling by heat conduction is relatively negligible with respect to the cooling by vapor shielding. In contrary, the vapor shielding even undesirably decreases when the dense strike point plasma sweeps away from the hot vaporizing surface region. Therefore, LMD seems not suitable in combination with this fast sweeping.

In addition, rare but a bit catastrophic unmitigated disruptions yield a vertical displacement event (VDE), depositing during a current quench huge plasma energy within 1-3 ms, predicted to yield significant damage of the divertor baffle. This proposed fast sweeping hardware may be also used for mitigating this catastrophic event, however, this was not yet studied.

## Summary

We demonstrated feasibility of a yet-never-tested concept aimed to suppress surface temperature rise during the undesired regular Edge Localised Mode, expelling plasma on tokamak divertor, predicted to highly exceed the tungsten damage threshold for any future thermonuclear reactors. Here proposed fast and far enough harmonic sweep of the strike point would decrease the temperature rise by a factor of $F_{\mathrm {STS}}$ = 3 on the EU DEMO reactor. This requires installation of a dedicated in-vessel 70 kg copper coil inside each 54 divertor cassettes, each in a resonant circuit with 18 kV capacitor and IGBT switch, requiring a MW-level power supply and other parameters in Table 2. Higher $F_{\mathrm {STS}}$ = 5 could be reached for $U_0=120$ kV with otherwise similar parameters, however, yielding unacceptably high 1.9 MJ dissipation per ELM.

Experimental confirmation of this concept on a current tokamak is needed. This technology might be usefully multiplied by other ELM-suppression techniques (namely the resonant magnetic perturbation^56^ and the impurity seeding^10^).

**Table 2:** The sweeping system parameters for the DEMO fusion tokamak.

CES	Capacitor energy storage		
$\frac{B_\text {coil}}{B_\text {COMSOL}}=\frac{I_\mathrm {coil}}{I_\mathrm {COMSOL}}=\sqrt{\frac{L_\text {COMSOL}}{L_\text {coil}}}=\frac{N_\text {COMSOL}}{N_\text {turns}}$	Rescaling from Fig. 4b to Fig. 4﻿c and Fig. 6		
$L=L_\text {CES}+L_\text {cabel}+L_\text {coil}$	Circuit inductance		
$C_\text {CES}^{-1}=(2\pi f_{\mathrm {swp}})^2\cdot L$	CES resonance capacitance		
$D=2\cdot D_\text {cable}+2\cdot N_\text {turns}\cdot 2\pi a$	Length of the copper Litz cable		
$m=\varrho _\text {Cu}\cdot D_\text {Litz}^2\cdot D$	Mass of the Litz cable		
$R_\mathrm {coil}=17\times 10^{-19}\cdot D\cdot D_\text {Litz}^{-2}$	Coil resistivity		
$E=\frac{1}{2}LI_0^2=\frac{1}{2}CU_0^2$	Stored magnetic and electric energy		
$\lambda _{\mathrm {swp}}=107$ mm$\cdot e^{-f/2.4\text {kHz}}\frac{N}{54}N_\text {turns}\cdot \frac{I_0}{\mathrm {MA}}$, Fig. 5	Magnetics-simulated strike point shift		
$F_\text {STS}=1 + \frac{5}{3}\ln (1+f_{\mathrm {swp}}\tau _{\mathrm {ELM}}^{\mathrm {decay}})\sqrt{\lambda _{\mathrm {swp}}/\lambda _q^{ELM}}$	Surface Temperature Suppression Factor: Fig. 2b		

**Assumed inputs**			
$D_{coil} + D_\mathrm {cable}$ length = 2*distance from tokamak to CES	330	m	
Divertor hole diameter (toroidal, vertical)	0.1, 0.3	m	
Coil outer diameter, length	0.3, 1	m	
In-vessel Volume of copper, coil, AC magnetic conductor	0.03, 0.07, 0.6	m$^3$	
Number of divertor cassettes & CES (each with 2 coils) $N=$	54		
DEMO ELM decay time $\tau _{\mathrm {ELM}}^{\mathrm {decay}}$^43^	3	ms	
DEMO ELM divertor decay length $\lambda _q^{ELM}$^43^	2	cm	
$C_\mathrm {cable}=1.6\times 10^{-11}D_\mathrm {cable}$ parasitic cable capacitance	1.6	nF	
$L_\mathrm {cable}=2.8\times 10^{-7}D_\mathrm {cable}$ parasitic cable inductance	28	$\upmu$H	
IGBT transistor switches for each coil	15 series	3 parallel	

**Study outputs**			
Voltage $U_0$ amplitude	± 18	± 120	kV
Optimal coil number of turns	63	100	
$\lambda _{\mathrm {swp}}$ swept strike point amplitude	±6	±16	cm
CES parasitic inductance $L_\text {CES}$	70	25	nH
the circuit parasitic resistance $R_\text {eff}=R_\mathrm {IGBT}+R_\mathrm {capacitor}+R_{coil}$	11	25	m$\Omega$
Capacity $C_\mathrm {CES}+C_\mathrm {cable}$	105	23	$\upmu$ F
$L=L_{capacitor}+L_{coil}+L_{cable}+L_{IGBT}$	0.14	0.3	*m*H
Resonant Sweep frequency $f_{\mathrm {swp}}=\frac{U_0}{2\pi \cdot L\cdot I_0}=(2\pi \sqrt{LC})^{-1}$	1.3	1.9	kHz
2*N* coils Ohmic losses $E^{\mathrm {tot}}_{\Omega \mathrm {/ELM}}=\frac{1}{2}R_\mathrm {eff}I_{\mathrm{coil}}^2\tau _\mathrm {ELM}=E_\text {eddy}+E^\text {RLC}_\text {circuit}$	0.22	2.2 !	MJ
AC Current $I_{\mathrm{coil}}$ amplitude	±16	±33	kA
Coil and Cable $D_\text {Litz}$ diameter	6	5	mm
Copper weight of 1 coil + cable	80	60	kg
Relative energy dissipation within 1 ELM $\frac{E^{\mathrm {tot}}_{\Omega \mathrm {/ELM}}}{E}$	0.24	0.25	
The predicted surface temperature suppression factor by the fast sweeping during an ELM $F_{\mathrm {STS}}$	3.1	5.1	

One coil, cable and CES (capacitor with IGBT switch) assumed in each 54 divertor cassettes. The coil number of turns is set in order to maximize $F_{\mathrm {STS}}$  from Fig. 6. Due to too high voltage and especially the total ohmic losses of the entire system, we consider as feasible only the 18 kV setup.

## Data Availability

The datasets and analyses details are available from the corresponding author on reasonable request.

## References

[1] Entler S (2018). Approximation of the economy of fusion energy. Energy.

[2] European Research Roadmap to the Realisation of Fusion Energy www.euro-fusion.org/eurofusion/roadmap

[3] Wenninger R (2017). The DEMO wall load challenge. Nucl. Fusion.

[4] Maviglia F (2020). Impact of plasma thermal transients on the design of the EU DEMO first wall protection. Fus. Eng. Des..

[5] Barrett, T., *et al.* Progress in the engineering design and assessment of the European DEMO First Wall and Divertor Plasma Facing Components. EUROFUSION CP(15)06/13. https://scipub.euro-fusion.org/wp-content/uploads/2015/11/EFCP150613.pdf

[6] https://financesonline.com/10-worlds-most-expensive-science-experiments/

[7] https://www.iter.org/sci/Goals

[8] Horacek J (2020). Scaling of L-mode heat flux for ITER and COMPASS-U divertors, based on five tokamaks. Nucl. Fusion.

[9] Sutton, G. P. & Biblarz O. Rocket propulsion elements (2017) 9th edition, Wiley, ISBN 9781118753910 https://www.vitalsource.com/products/rocket-propulsion-elements-george-p-sutton-oscar-v9781118753910

[10] Ravensbergen T (2021). Real-time feedback control of the impurity emission front in tokamak divertor plasmas. Nat. Commun..

[11] Pitts RA (2019). Physics basis for the first ITER tungsten divertor. Mater. Energy.

[12] Jiang M (2022). Inhibiting the Leidenfrost effect above 1000°C for sustained thermal cooling. Nature.

[13] Horacek J (2021). Predictive modelling of liquid metal divertor: From COMPASS tokamak towards Upgrade. Phys. Scr..

[14] Panek R (2017). Conceptual design of the COMPASS upgrade tokamak. Fusion Eng. Des..

[15] Vondracek P (2021). Preliminary design of the COMPASS upgrade tokamak. Fusion Eng. Des..

[16] Weinzettl V (2019). Constraints on conceptual design of diagnostics for the high magnetic field COMPASS-U tokamak with hot walls. Fusion Eng. Des..

[17] Coda S (2019). Physics research on the TCV tokamak facility: From conventional to alternative scenarios and beyond. Nucl. Fusion.

[18] Reimerdes H (2020). Assessment of alternative divertor configurations as an exhaust solution for DEMO. Nucl. Fusion.

[19] Loarte A (2006). Chaos cuts ELMs down to size. Nat. Phys..

[20] Cruz N (2018). On the control system preparation for ELM pacing with vertical kicks experiments at TCV. Fusion Eng. Des..

[21] Evans T (2006). Edge stability and transport control with resonant magnetic perturbations in collisionless tokamak plasmas. Nat. Phys..

[22] Lennholm M (2021). Statistical assessment of ELM triggering by pellets on JET. Nucl. Fusion.

[23] Eich T (2017). ELM divertor peak energy fluence scaling to ITER with data from JET, MAST and ASDEX upgrade. Nuclear Mater. Energy.

[24] Adamek J (2017). Electron temperature and heat load measurements in the COMPASS divertor using the new system of probes. Nucl. Fusion.

[25] Suslova A (2014). Recrystallization and grain growth induced by ELMs-like transient heat loads in deformed tungsten samples. Nat. Sci. Rep..

[26] Coenen J (2015). ELM induced tungsten melting and its impact on tokamak operation. J. Nuclear Mater..

[27] Coenen J (2015). ELM-induced transient tungsten melting in the JET divertor. Nucl. Fusion.

[28] Kuang AQ (2020). Divertor heat flux challenge and mitigation in SPARC. J. Plasma Phys..

[29] Maviglia F (2021). Impact of plasma-wall interaction and exhaust on the EU-DEMO design. Nuclear Mater. Energy.

[30] M. Komm *et al.* Observations of ELM buffering in argon and nitrogen seeded discharges in AUG. Presentation at the 25th International Conference on Plasma Surface Interactions in Controlled Fusion Devices. https://www.psi2022.kr/program/program_02.html?searchTxt=komm

[31] Gunn JP (2017). Ion orbit modelling of ELM heat loads on ITER divertor vertical targets. Nuclear Mater. Energy.

[32] Arnoux G (2015). Thermal analysis of an exposed tungsten edge in the JET divertor. J. Nuclear Mater..

[33] Krieger K (2017). Investigation of transient melting of tungsten by ELMs in ASDEX upgrade. Phys. Scr..

[34] Silvagni D (2020). I-mode pedestal relaxation events at ASDEX Upgrade. Nucl. Fusion.

[35] Horacek J (2018). Plans for liquid metal divertor in tokamak compass. Plasma Phys. Rep..

[36] Horacek J (2020). Modeling of COMPASS tokamak divertor liquid metal experiments. Nuclear Mater. Energy.

[37] Kallenbach A (2021). Developments towards an ELM-free pedestal radiative cooling scenario using noble gas seeding in ASDEX Upgrade. Nuclear Fusion.

[38] Jacquinot J (1999). Deuterium-tritium operation in magnetic confinement experiments: Results and underlying physics. Plasma Phys. Control. Fusion.

[39] Romanelli F (2013). Overview of the JET results with the ITER-like wall. Nucl. Fusion.

[40] Clery D (2022). European fusion reactor sets record for sustained energy. Science.

[41] Putterich T (2010). Calculation and experimental test of the cooling factor of tungsten. Nucl. Fusion.

[42] Wang Shuming (2020). Thermal damage of tungsten-armored plasma-facing components under high heat flux loads. Nat. Sci. Rep..

[43] Horacek J (2017). Feasibility study of fast swept divertor strike point suppressing transient heat fluxes in big tokamaks. Fusion Eng. Des..

[44] Li M, Maviglia F, Federici G, You J-H (2016). Sweeping heat flux loads on divertor targets: Thermal benefits and structural impacts. Fusion Eng. Des..

[45] Lukes, S. Horacek, J. Fast swept divertor suppressing transient heat pulses in tokamaks. Master Thesis, Czech Technical University (2022). https://dspace.cvut.cz/handle/10467/100839

[46] You JH (2017). Progress in the initial design activities for the European DEMO divertor: Subproject “Cassette”. Fusion Eng. Des..

[47] Pitts RA (2017). Physics conclusions in support of ITER W divertor monoblock shaping. Mater. Energy.

[48] Gunn JP (2017). Surface heat loads on the ITER divertor vertical targets. Nucl. Fusion.

[49] https://en.m.wikipedia.org/wiki/Solenoid

[50] DEMO Eurofusion private document. https://idm.euro-fusion.org

[51] Adamek J (2020). On the transport of edge localized mode filaments in the tokamak scrape-off layer. Nuclear Fusion.

[52] Hamalainen H (2014). AC resistance factor of Litz-Wire windings used in low-voltage high-power generators. IEEE Trans. Ind. Electron..

[53] Gaudreau, M. *et al * Solid-state power systems for pulsed electric field (PEF) processing. In *2005 IEEE Pulsed Power Conference Solid-State Power Systems for Pulsed Electric Field (PEF) Processing*. 10.1109/PPC.2005.300605

[54] Fridman B (2011). A 0.5-MJ 18-kV module of capacitive energy storage. IEEE Trans. Plasma Sci..

[55] Ma K (2015). Evaluation and design tools for the reliability of wind power converter system. J. Power Electron..

[56] Becoulet M (2012). Screening of resonant magnetic perturbations by flows in tokamaks. Nucl. Fusion.

[57] Dejarnac R (2020). Overview of COMPASS tokamak divertor liquid metal experiments. Nuclear Mater. Energy.

